# Enhanced short chain fatty acids production from waste activated sludge conditioning with typical agricultural residues: carbon source composition regulates community functions

**DOI:** 10.1186/s13068-015-0369-x

**Published:** 2015-11-25

**Authors:** Zechong Guo, Aijuan Zhou, Chunxue Yang, Bin Liang, Thangavel Sangeetha, Zhangwei He, Ling Wang, Weiwei Cai, Aijie Wang, Wenzong Liu

**Affiliations:** State Key Laboratory of Urban Water Resource and Environment, Harbin Institute of Technology (SKLUWRE, HIT), Harbin, China; College of Environmental Science and Engineering, Taiyuan University of Technology, Taiyuan, China; Key Laboratory of Environmental Biotechnology, Research Center for Eco-Environmental Sciences, Chinese Academy of Sciences, Beijing, China

**Keywords:** Waste activated sludge (WAS), Biomass conditioning, Short-chain fatty acids (SCFAs), Microbial community shift, Agricultural residues (ARs)

## Abstract

**Background:**

A wide range of value-added by-products can be potentially produced from waste activated sludge (WAS) through anaerobic fermentation, among which short-chain fatty acids (SCFAs) are versatile green chemicals, but the conversion yield of SCFAs is usually constrained by the low carbon-to-nitrogen ratio of the original WAS. Conditioning of the WAS with cellulose-containing agricultural residues (ARs) has been reported to be an efficient and economical solution for balancing its nutrient components. However, contributions of different ARs to SCFAs production are still not well understood.

**Results:**

To optimize SCFAs production through carbon conditioning of WAS, we investigated the effects of two typical ARs [straws and spent mushroom substrates (SMSs)] on WAS hydrolysis and acidification in semi-continuous anaerobic fermentation. Straw-conditioning group showed a threefold increase in short-chain fatty acids yield over blank test (without conditioning), which was 1.2-fold higher than that yielded by SMS-conditioning. The maximum SCFAs yield in straw-conditioning groups reached 486.6 mgCOD/gVSS (Sludge retention time of 8 d) and the highest volumetric SCFAs productivity was 1.83 kgCOD/(${\text{m}}_{\text{reactor}}^{3} \cdot {\text{d}}$) (Sludge retention time of 5 d). In batch WAS fermentation tests, higher initial SCFAs production rates were achieved in straw-conditioning groups [49.5 and 52.2 mgCOD/(L·h)] than SMS-conditioning groups [41.5 and 35.2 mgCOD/(L·h)]. High-throughput sequencing analysis revealed that the microbial communities were significantly shifted in two conditioning systems. Carbohydrate-fermentation-related genera (such as *Clostridium IV, Xylanibacter*, *and Parabacteroides*) and protein-fermentation-related genus *Lysinibacillus* were enriched by straw-conditioning, while totally different fermentation genera (*Levilinea*, *Proteiniphilum*, and *Petrimonas*) were enriched by SMS-conditioning. Canonical correlation analysis illustrated that the enrichment of characteristic genera in straw-conditioning group showed positive correlation with the content of cellulose and hemicellulose, but showed negative correlation with the content of lignin and humus.

**Conclusions:**

Compared with SMSs, straw-conditioning remarkably accelerated WAS hydrolysis and conversion, resulting in higher SCFAs yield. Distinct microbial communities were induced by different types of ARs. And the communities induced by straw-conditioning were verified with better acid production ability than SMS-conditioning. High cellulose accessibility of carbohydrate substrates played a crucial role in enriching bacteria with better hydrolysis and acidification abilities.

**Electronic supplementary material:**

The online version of this article (doi:10.1186/s13068-015-0369-x) contains supplementary material, which is available to authorized users.

## Background

Nowadays, WAS, the main solid waste product from the wastewater treatment process, is considered as a valuable biomass resource and is gaining worldwide attentions [1, 2]. SCFAs production from anaerobic fermentation of WAS has been proven to be a feasible and effective carbon resource recovery process [3–5]. Compared with time-consuming conventional sludge digestion processes for biogas (generally 20 ~ 30 d for a single batch), SCFAs-producing processes with proper pretreatments are completed in a relatively short operation cycle (5 ~ 8 d) [6–9]. Moreover, SCFAs have been recognized as high value-added green chemicals, which can be used for enhancing biological nutrient removal from wastewater [10, 11], polyhydroxyalkanoates production [12], hydrogen or methane production by microbial electrolysis cell (MEC) [13–15], and so on.

Nevertheless, SCFAs yield is generally limited by the unbalanced nutrient component, especially the low carbon-to-nitrogen ratio (C/N ratio) [16]. This unbalanced ratio results in the inefficient conversion of complex organic matters in WAS, and conditioning or co-fermentation with carbon-rich ARs has been reported as a cost-effective solution for this issue. A 69 % increase in SCFAs yield was obtained by co-digestion of corn straw and WAS than that produced by sludge alone [17]. A maximal SCFAs yield of 712 mgCOD/gVSS was obtained by coupling sludge pretreatments with Agaricus bisporus conditioning [18].

It is interesting to note that, in these literatures, adjusting the C/N ratio of fermentation system to the same level (20/1) with different kinds of external carbohydrate substrates led to enormous variations in acid production promoting effects (12 times increase over sole sludge with perennial ryegrass, but only 1.4 times with bagasse) [19, 20]. This strongly implied that the effect of carbon conditioning is more than just C/N ratio adjustment. It is crucial to investigate the vital roles of the external carbohydrate substrates in enhancing WAS fermentation and the different effects that will be produced when using different ARs as external carbohydrate substrates.

Recent researches have portrayed that the presence of some recalcitrance ingredients like lignin would lead to a reduction of the degradability of lignocellulosic feedstocks [21, 22], and this in turn may affect the composition and metabolism activity of fermentation bacteria. In order to gain a basic understanding of screening standard for suitable external carbohydrate substrates for WAS conditioning, it is important to know the effects of agricultural waste composition on WAS fermentation and related functional microbial community structures.

In this study, SCFAs production from WAS fermentation conditioned by two typical types of lignocellulosic ARs, straws and SMSs, was investigated by semi-continuous fermentation experiments. After a long-term operation, sludge in different conditioning systems from the semi-continuous reactors was examined for batch WAS fermentation tests and high-throughput sequencing analysis. Batch WAS fermentation tests were carried out to determine the acid-producing ability of acclimated bacteria in different conditioning systems in the absence of carbohydrate substrates, and high-throughput sequencing analysis was conducted to probe the microbial community structure shifts and functional community evolutions induced by different ARs conditioning. The effects of different types of ARs on WAS hydrolysis and SCFAs production were analyzed, and the linkage between microbial community shifts and ARs compositions was thoroughly discussed.

## Results

### SCFAs production of WAS conditioned by different ARs

**Fig. 1:**
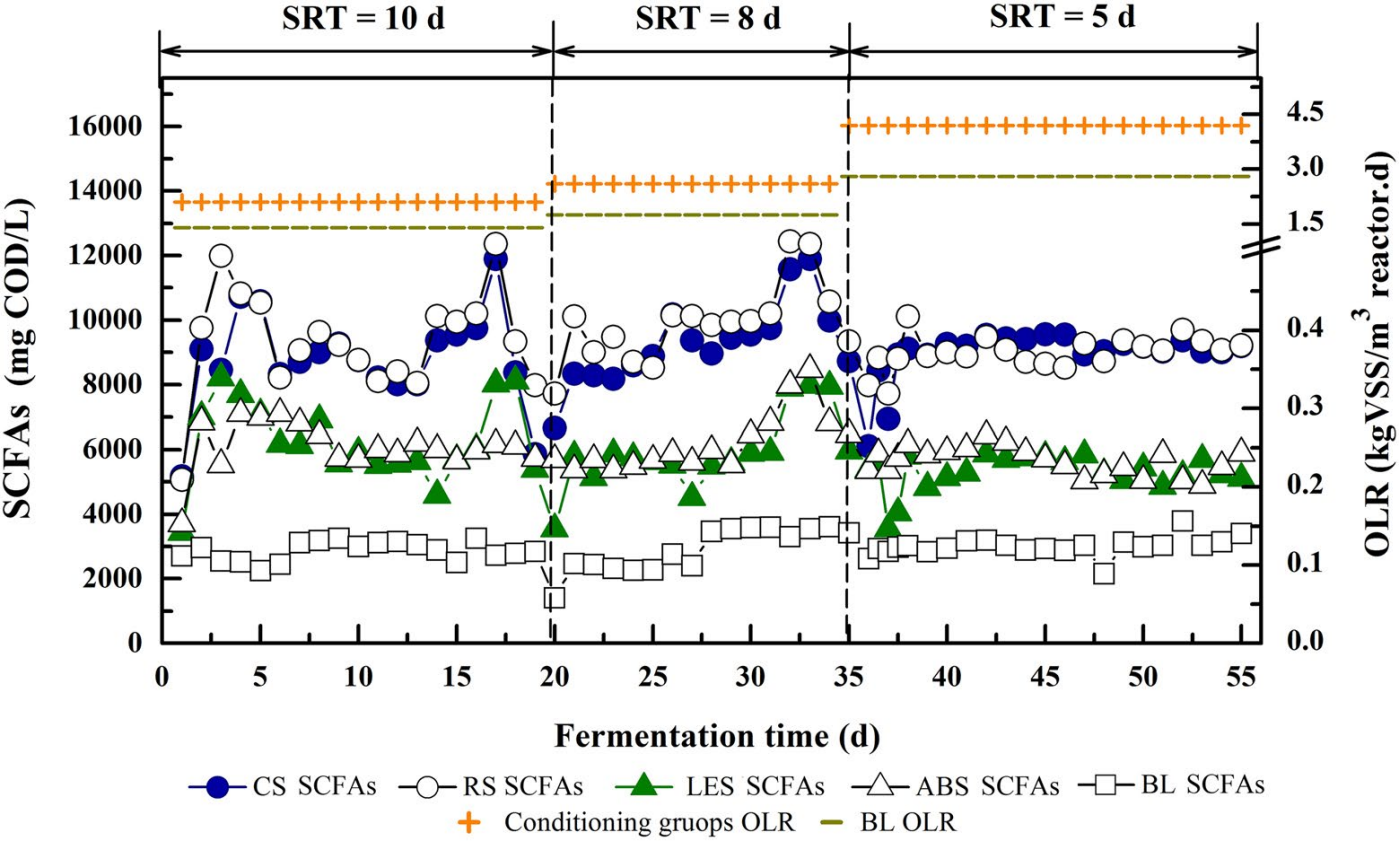
Effect of typical AR-conditioning on SCFAs production during different SRT operation stages. The figure shows the concentrations of SCFAs in CS, RS, LES, ABS, and BL group during operation stages of SRT 10 d, SRT 8 d, and SRT 5 d. The OLR of AR-conditioning groups and BL under each stage were also given in the figure

Considering the significant similarities between corn straw (CS) and rice straw (RS), lentinus edodes substrate (LES), and agaricus bisporus substrate (ABS) in acid-producing performance during the whole fermentation process (an additional word file shows the detailed statistical analysis results, see Additional file 1), CS and RS were mentioned as straw-conditioning groups, whereas LES and ABS as SMS-conditioning groups in the following discussion. All AR-conditioning groups were stable and showed remarkable SCFAs production during the operation process, along with significant superiorities over the blank (BL) (Fig. 1). The highest level of SCFAs was detected at a sludge retention time (SRT) of 8 d for all reactors, which was 12 ~ 22 % higher than those at SRT of 10 and 5 d. On SRT 8 d, the average SCFAs concentrations of straw-conditioning groups were up to 10217.2 mgCOD/L, and SMS-conditioning groups were 6436.7 mgCOD/L, while BL was only 3508.6 mgCOD/L. Although the total organic carbon values (mgC/gVSS) were adjusted to a similar level in all tests, a distinct difference was shown in acid-producing capabilities between two types of ARs. Generally, straw-conditioning groups showed a 3.0-fold increase over BL, while SMS-conditioning groups only showed a 1.8-fold increase. Apparently, conditioning with straws was more beneficial for SCFAs production than conditioning with SMSs.

In each stage, SCFAs concentration in all groups apparently shifted to relatively stable levels in spite of the initial fluctuation for several days (Fig. 1). In straw-conditioning groups, SCFAs yield was 423.5 mgCOD/gVSS for SRT of 10 d with an organic load rate (OLR) of 2.1 kgVSS/(m^3^·d). When SRT was shortened to 8 d [OLR 2.6 kgVSS/(m^3^·d)], the SCFAs yield increased by 15 % and reached the maximum value of 486.6 mgCOD/gVSS. The increase of SCFAs yield was slightly lower (12 %) in SMS-conditioning groups when SRT was shortened from 10–8 d, and the maximum yield was 306.5 mgCOD/gVSS. When SRT was further shortened to 5 d, in spite of a slight decrease in SCFAs yields, all groups reached the maximum volumetric SCFAs productivity of 1.83 and 0.98 kgCOD/(${\text{m}}_{\text{reactor}}^{3} \cdot {\text{d}}$) for straw-conditioning groups and SMS-conditioning groups due to the substantial increase of OLR [from 2.6–4.2 kgVSS/(m^3^·d)].

**Fig. 2:**
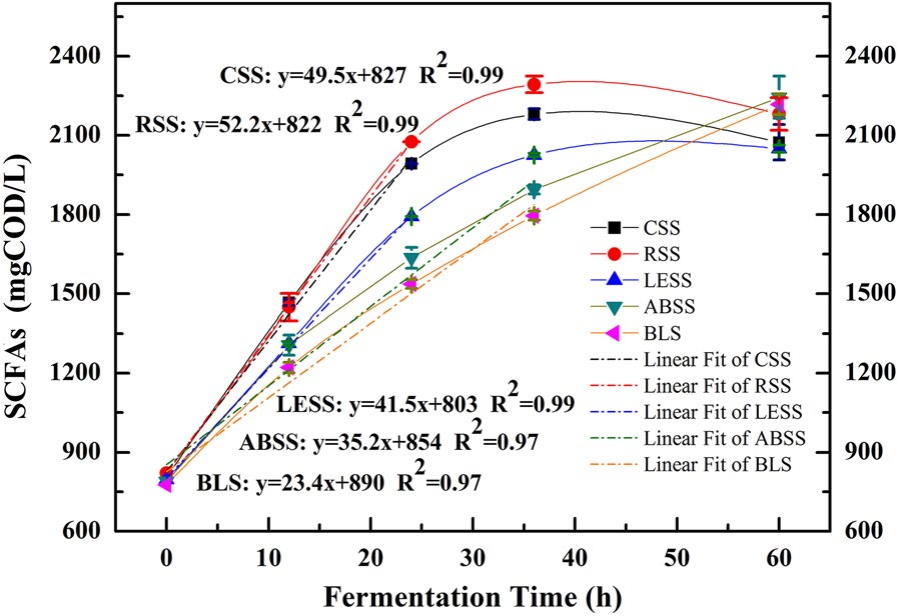
SCFAs production performance of batch WAS fermentation tests. The variation of SCFAs concentrations during the fermentation time in group CSS, RSS, LESS, ABSS, and BLS in batch WAS fermentation tests are shown in this figure. Linear fit analysis was applied; formula and correlation coefficient of each *line* are portrayed in the figure

Batch WAS fermentation tests were carried out to determine the SCFAs-producing ability of anaerobic bacteria, which were acclimated in different AR-conditioning fermentation systems without the influence of external carbon substrates. SCFAs concentrations of group CSS, RSS, and LESS were increasing linearly (*R*^2^ = 0.99) in first 24 h, and reached their maximum values of 2180, 2294, and 2025 mgCOD/L at about 36 h, while ABSS and BLS followed a linearly upward trend for whole 60 h with maximum values of 2244.3 and 2217.7 mgCOD/L (Fig. 2). Linear regression analysis was applied to obtain the initial SCFAs production rates. The initial SCFAs production rates of CSS, RSS, LESS, and ABSS were 49.5, 52.2, 41.5 and 35.2 mgCOD/(L·h), respectively. They had increased 111, 123, 77,and 50 %, respectively, compared with BLS [23.4 mgCOD/(L·h)]. The bacteria in straw-conditioning fermentation system were proven to have better ability in degrading WAS and producing SCFAs than those in SMS-conditioning fermentation system.

### WAS hydrolysis performance

**Fig. 3:**
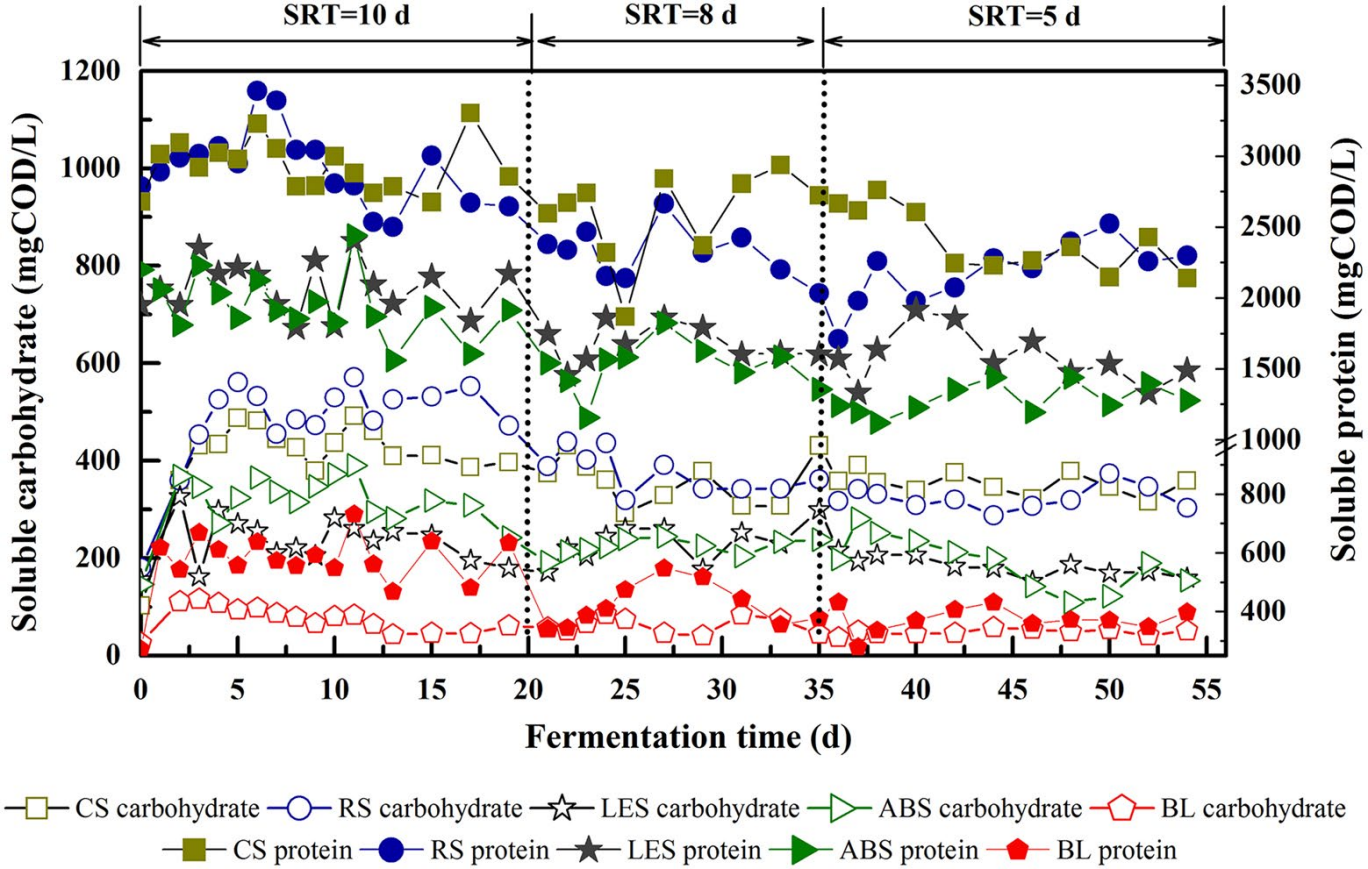
Effect of AR-conditioning on soluble substrates concentration during different SRT operation stages. The figure showed the variation of soluble carbohydrate and soluble protein concentrations during operation stages of SRT 10 d, SRT 8 d, and SRT 5 d in group CS, RS, LES, ABS, and BL

Consistent with SCFAs production performance, straw-conditioning groups also showed significant advantages over SMS-conditioning groups on the hydrolysis of complex organic matters and the release of soluble carbohydrate and protein (Fig. 3). Compared with BL (48.1 mgCOD/L SRT = 5 d), the concentration of soluble carbohydrate in straw-conditioning groups increased 7.0-fold (338.6 mgCOD/L), while SMS-conditioning groups had a 3.9-fold increase (187.3 mgCOD/L). The soluble protein concentration of straw-conditioning groups and SMS-conditioning groups increased 6.1-fold (2292.2 mgCOD/L SRT = 5 d) and 3.8-fold (1431.8 mgCOD/L), respectively, compared with BL (372.7 mgCOD/L). Correspondingly, volatile suspended solids (VSS) removal in straw-conditioning groups (7.6 g/L SRT = 5 d) was also higher than that in SMS-conditioning groups (6.0 g/L SRT = 5 d) (Table 1). It is well known that the hydrolysis of particulate organic matters was generally a rate-limiting step in the anaerobic fermentation process of complex solid substrates (like WAS) [23, 24]. So the enhancement in soluble carbohydrate and protein release was supposed to contribute to the increase of SCFAs production.

**Table 1:** Performance of semi-continuous reactors for CS, RS, LES, ABS, and BL

Parameter	Stage 1 SRT = 10 d					Stage 2 SRT = 8 d					Stage 3 SRT = 5 d				
	CS	RS	LES	ABS	BL	CS	RS	LES	ABS	BL	CS	RS	LES	ABS	BL
OLR^a^	2.1				1.4	2.6				1.8	4.2				2.8
SCFAs^b^	8743.1	9044.2	5576.4	5880.4	2918.6	9942.7	10,491.8	6271.9	6601.6	3508.6	9039.0	9247.2	4859.0	4899.0	2880.1
Product yield^c^	416.3	430.7	265.5	280.0	208.5	473.5	499.6	298.7	314.4	250.6	430.4	440.3	231.4	233.3	137.1
Acetate^d^	38.2	39.1	41.7	47.5	47.3	43.2	45.5	54.6	54.1	51.3	48.1	46.0	52.2	58.9	53.5
Propionate^d^	25.3	22.5	21.1	18.4	13.2	31.8	26.3	24.1	20.2	15.4	25.8	22.3	22.5	20.1	16.3
Other SCFAs^d^	36.5	38.4	37.2	34.1	39.5	25.0	28.2	21.3	25.7	34.3	26.1	31.7	25.3	21.0	30.2
Soluble chemical oxygen demand (SCOD)^e^	13,428.1	14,039.2	9742.8	9961.8	3778.9	12,361.2	12,528.5	9233.9	8761.2	3461.5	11,340.3	12,263.1	8545.5	8025.3	3749.3
Soluble carbohydrate^b^	430.1	495.1	232.1	299.6	70.4	343.8	362.4	246.5	229.8	63.9	353.6	323.5	184.2	190.5	48.1
Soluble protein^b^	2893.1	2852.9	2033.2	1881.4	553.2	2554.3	2338.5	1708.6	1544.3	433.3	2405.2	2179.2	1578.8	1284.7	372.7
NH_4_^+^-N^e^	508.5	521.2	354.2	350.6	130.4	455.0	488.5	247.5	298.5	106.6	525.6	490.3	232.8	217.3	76.1
PO_4_^3−^-P^e^	161.4	161.3	130.1	103.9	42.7	134.8	159.5	103.2	85.2	35.3	145.7	163.2	112.3	82.5	33.4
Total suspended solids (TSS) decrease^f^	9.8	10.1	8.3	6.6	3.3	9.2	8.9	8.3	7.2	2.9	8.1	8.5	7.4	6.1	2.8
Volatile suspended solids (VSS) removal^f^	9.2	9.1	7.3	6.2	2.9	8.6	8.5	7.6	5.8	2.5	7.5	7.8	6.9	5.2	2.4

All numbers were the average values of all valid data

^a^Unit is kgVSS/m^3^/d; ^b^unit is mgCOD/L; ^c^unit is mgCOD/gVSS; ^d^percentage of specific VFA in total SCFAs,  %; ^e^unit is mg/L; ^f^the decrease amount of TSS and VSS, unit is g/L

The increase of soluble carbohydrate was mainly resulted from the additional carbohydrates provided by ARs. But the increase of soluble protein was mainly caused by the enhanced hydrolysis of WAS instead of ARs, because of the extremely low concent of protein in ARs (2.9, 0.8, 4.6, and 6.9 % for CS, RS, LES, and ABS, respectively).

Besides the hydrolysis of particulate substrates, the degradation and conversion of carbohydrate and protein were also enhanced in the AR-conditioning groups (especially in straw-conditioning groups), which can be inferred from the increase of propionic acid (HPr) and ammonia production (Table 1). The yield coefficients of HPr from monosaccharides and amino acids (i.e., ƒ_pro,su_ and ƒ_pro,aa_) postulated by Anaerobic Digestion Model No.1 (ADM1) were 0.27 and 0.05, respectively [25], which indicated that more HPr could be produced from carbohydrate conversion than from equivalent protein conversion. In BL, the percentage of HPr was 15 % (SRT 8 d); in SMS-conditioning groups the percentage was promoted to 22 %, and in straw-conditioning groups it was further elevated to 28 %. Ammonia, mainly produced from the degradation of amino acids, was closely related to the conversion of protein to SCFAs. Concentrations of ammonia increased 4.5-fold in straw-conditioning groups but only 2.5-fold in SMS-conditioning groups.

The increase of OLR led to downward trends in soluble substrate concentrations for all groups (Fig. 3). But it was interesting to note that the promotion rate of soluble protein concentration (compared with BL) in straw-conditioning groups increased from 5.2-fold to 6.1-fold when SRT was shortened from 10–5 d (Table 1), which demonstrated that the predominance of AR-conditioning (especially the straw-conditioning) increased with the increase of OLR. This clearly indicated that AR-conditioning would be of benefit for process stability under short SRTs.

### Microbial community shift under different ARs conditioning

**Fig. 4:**
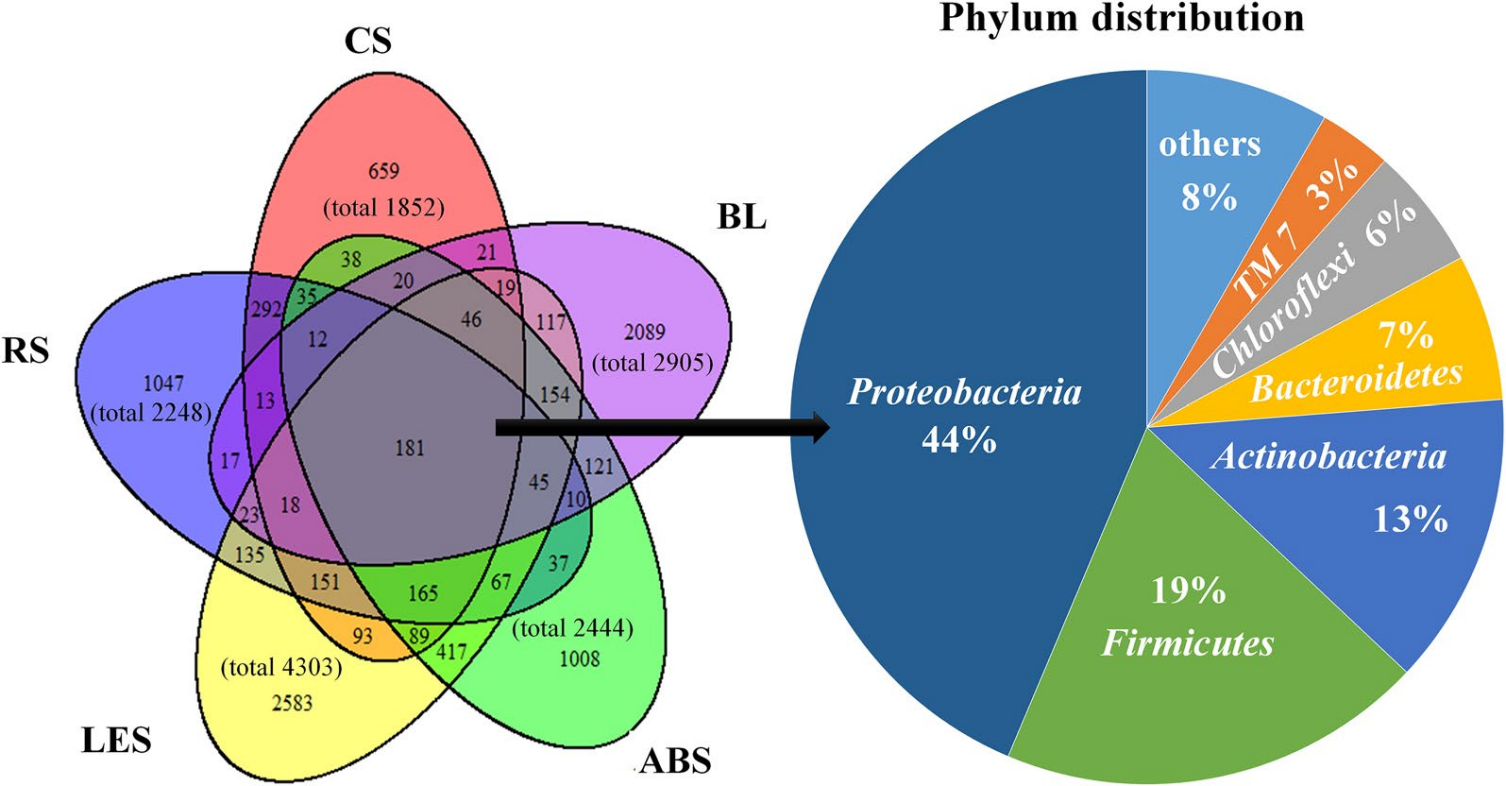
The overlapping of classified OTUs and the bacteria phylum distribution of overlapped part. The overlapping nature of classified OTUs from CS, RS, LES, ABS, and BL was shown in the figure, and the distribution of overlap part at phylum level was displayed

**Fig. 5:**
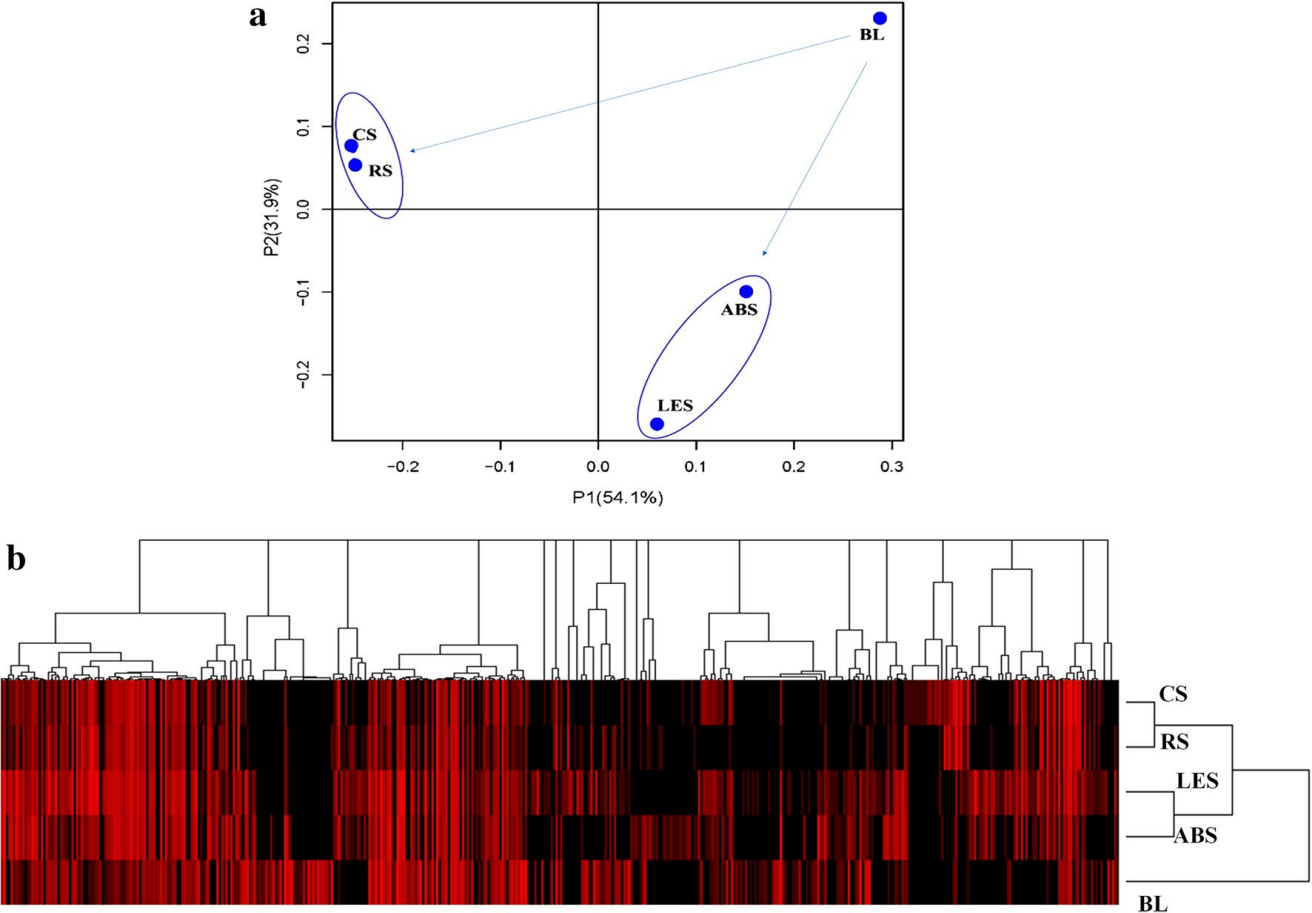
Principal component analysis and hierarchical clustering analysis. **a** Showed the result of principal component analysis (PCA) of classified OTUs from CS, RS, LES, ABS, and BL; **b** showed the result of hierarchical clustering analysis of classified OTUs from CS, RS, LES, ABS, and BL

The total classified operational taxonomic units (OTUs) in five bacteria communities were 9721, but only 181 OTUs (1.8 %) were shared by all samples (Fig. 4), and the shared OTUs mainly belonged to phylum *Proteobacteria* (44 %), *Firmicutes* (19 %), and *Actinobacteria* (13 %). CS and RS shared 867 OTUs (46.8 % of CS, 38.5 % of RS); LES and ABS shared 1164 OTUs (27 % of LES, 47.6 % of ABS). Although all AR-conditioning groups’ C/N ratios were quite similar (12/1, 13/1, 12/1, and 9/1 for CS, RS, LES, and ABS, respectively), their microbial community structures were significantly grouped by the types of ARs. Principal component analysis (PCA) of classified OTUs in five samples revealed that the microbial communities in ARs conditioning groups were significantly shifted from that in BL (Fig. 5a). Relatively similar communities occurred in groups CS-RS and LES-ABS, but were totally non-similar with each other. This was further proven by the results of hierarchical clustering analysis (Fig. 5b).

The Shannon–Weaver index of straw-conditioning groups (4.6 for CS and 4.8 for RS) was obviously lower than other groups (5.8 for LES, 5.5 for ABS, and 5.7 for BL), and so were the Simpson Index and Richness (Table 2). It illustrated that the microbial community diversity of straw-conditioning groups was significantly reduced, which inferred that functional bacteria populations were enriched in the microbial communities of straw-conditioning groups.

**Table 2:** Alpha diversity of microbial community in each sample

Sample	Shannon index	Simpson index	Richness
CS	4.6	18.2	1852
RS	4.8	19.5	2248
LES	5.8	66.7	4303
ABS	5.4	48.5	2444
BL	5.6	53.3	2905

**Fig. 6:**
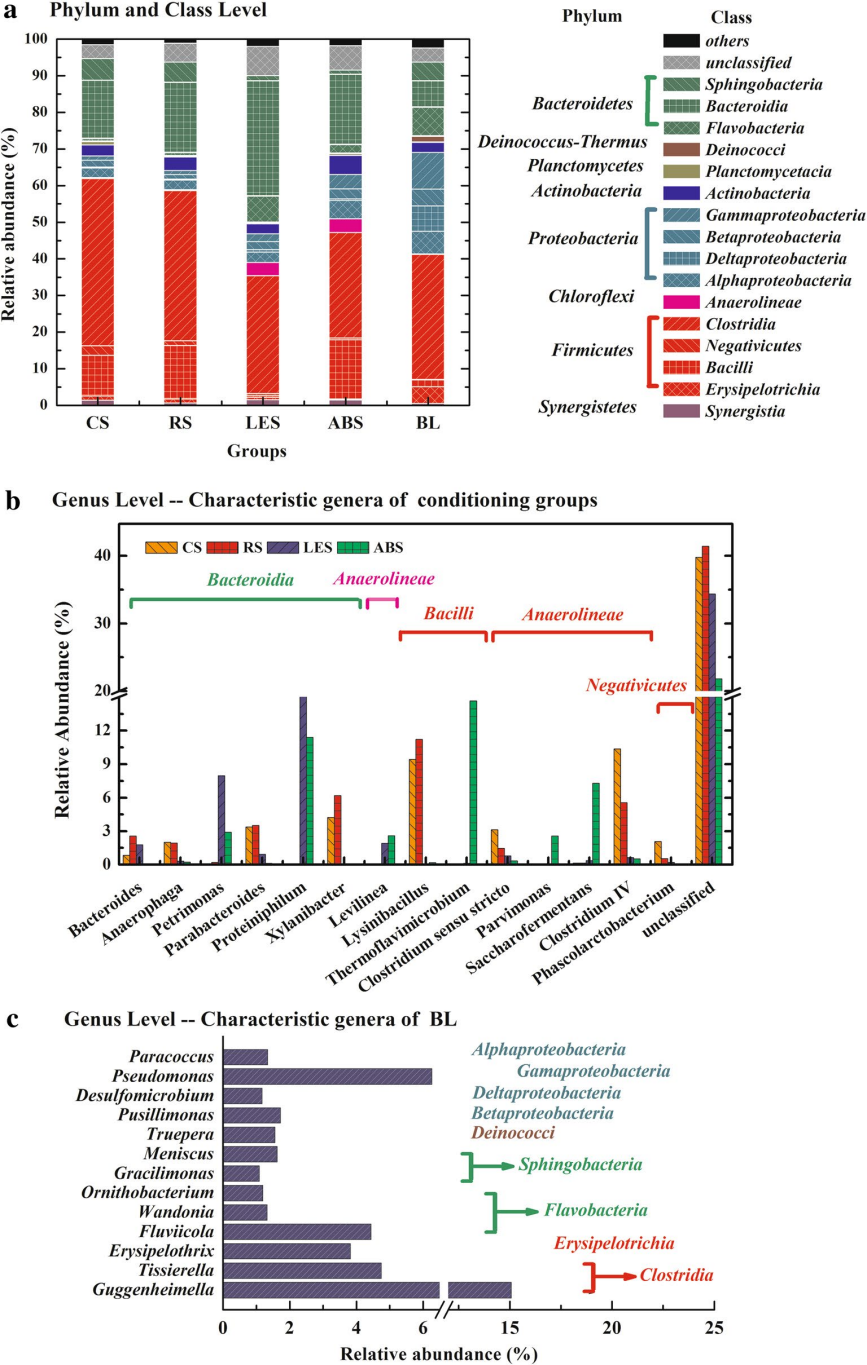
Taxonomical classification of sequences. **a** Showed the taxonomic classification of sequences from bacterial communities of CS, RS, LES, ABS, and BL at the phylum and class level; **b** illustrated the relative abundance of characteristic genera in group CS, RS, LES, and ABS and c depicted the relative abundance of characteristic genera in BL

The distributions of bacteria at phyla, class, and genera level further explained the differences among straw-conditioning groups, SMS-conditioning groups, and BL in detail. Five sludge samples showed extremely high diversities with a total of 32 identified phyla and 58 classes observed. The distribution of the main 16 bacteria classes (relative abundance >1 % in at least one sample) and their corresponding phyla were shown in Fig. 6a. The phyla *Firmicutes, Bacteroidetes*, and *Proteobacteri*a, which were recognized as common fermentation phyla, were dominant in all five communities with a total proportion of 91.6, 92.2, 84.1, 84.0, and 90.6 % in CS, RS, LES, ABS, and BL, respectively. But the distribution of three phyla in five samples presented an obvious difference. *Firmicutes* had the highest relative abundance in straw-conditioning groups (about 60 %), followed by ABS (48.3 %), but only 41.1 % in BL. LES, as an exception, had the most *Bacteroidetes* and much fewer *Firmicutes. Proteobacteri*a decreased in all conditioning samples, from 28.3 % in BL to 6.0 % in CS, 5.3 % in RS, 7.9 % in LES, and 12.1 % in ABS. At class level, *Bacteroidia* (phylum *Bacteroidetes*), *Bacilli* (phylum *Firmicutes*), and *Clostridia* (phylum *Firmicutes*) substantially increased. But *α*-*, β*-*, δ*-*, γ*-*proteobacteria* (phylum *Proteobacteria*) and *Erysipelotrichia* (phylum *Firmicutes*) decreased in AR-conditioning groups, especially in straw-conditioning groups.

The information of genera with relative abundance >1 % in at least one sample were listed in an additional word file (see Additional file 2). The characteristic genera shared by two straw-conditioning samples and genera shared by two SMS-conditioning samples were shown in Fig. 6b. The dominant genera in CS and RS mainly consisted of *Lysinibacillus* (10 %), *Clostridium IV* (6 ~ 10 %), *Xylanibacter* (5 %), *Parabacteroides* (3.5 %), *Clostridium sensustricto* (2 %), and *Anaerophaga* (2 %), while these genera were quite rare in SMS-conditioning samples (below 1 %) and BL sample (below 0.2 %). The dominant genera shared by LES and ABS included *Proteiniphilum* (11 ~ 17 %), *Petrimonas* (3 ~ 8 %), *Levilinea* (2 %), and *Tissierella* (2 %), and their prevalence was quite low in CS and RS (below 0.1 %). Enrichment of *Lysinibacillus*, *Clostridium IV*, and *Xylanibacter* better explained the increase of class *Bacilli*, *Clostridia*, and *Bacteroidia* in CS and RS. Comparatively, enrichment of *Proteiniphilum* and *Petrimonas* was the cause of increase of class *Bacteroidia* in LES and ABS. On the whole, the communities in straw-conditioning groups predominantly contributed to carbohydrate and protein fermentation, which was indicated by a reduced diversity shown in Shannon–Weaver index of 4.6 for CS, 4.8 for RS, 5.8 for LES, 5.5 for ABS, and 5.7 for BL (Table 2).

The dominant genera in BL included *Guggenheimella* (15.1 %), *Pseudomonas* (6.3 %), *Saccharofermentans* (5.6 %), *Tissierella* (4.8 %), *Fluviicola* (4.4 %), *Erysipelothrix* (3.8 %), *Proteiniphilum* (3.6 %), *Petrimonas* (2.8 %), and so on (Fig. 6c). Among these genera, *Proteiniphilum* and *Petrimonas* were enriched in SMS-conditioning samples as two most abundant genera, and *Guggenheimella* (2.3 %), *Saccharofermentans* (7.3 %), and *Tissierella* (2 %) remained in ABS to a lesser content, whereas the other genera sharply decreased in AR-conditioning samples (below 0.5 %).

**Fig. 7:**
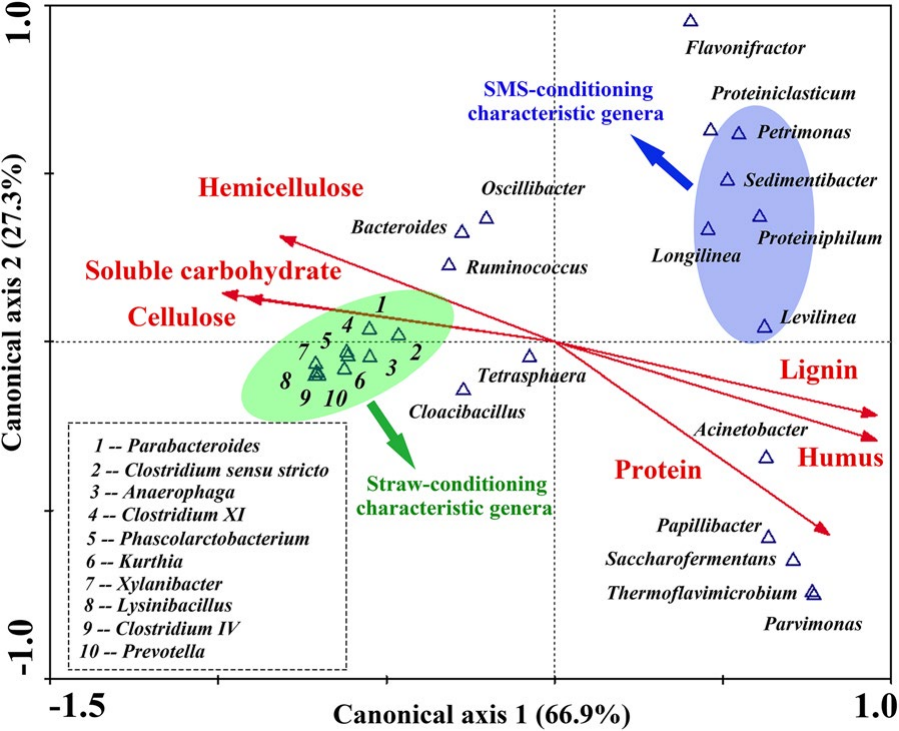
Canonical correlation analysis between enriched genera enrichment and main compositions in ARs. The figure showed the result of canonical correlation analysis (CCA) between characteristic genera in AR-conditioning groups and the content of cellulose, hemicelluloses, soluble carbohydrate, protein, lignin, and humus in ARs

The relationship between characteristic genera in AR-conditioning groups and main compositions in ARs was explained by canonical correlation analysis (CCA) (Fig. 7). The contents of lignin, humus, and protein were proven to be positively correlated with the first canonical axis (explained 66.9 % of the variance of genera distribution), and the contents of cellulose, soluble carbohydrate, and hemicellulose showed negative interrelations. For axis 2 (explained 27.3 % variance), only protein content showed good negative correlations. The detailed information was shown in an additional word file (see Additional file 3). The characteristic genera of straw-conditioning groups were *Parabacteroides, Lysinibacillus, Clostridium IV, Xylanibacter*, *Prevotella,* etc. They were all located on negative axle of axis 1, indicating that these genera could be enriched by the feedstocks with high content of cellulose, hemicellulose and their hydrolysates, and with limited content of lignin and humus. When the content of lignin and humus increased, the dominant genera in SMS-conditioning groups like *Petrimonas, Sedimentibacter, Proteiniphilum*, and *Levilinea* were more likely to be enriched. The intersection angle between protein and axis 1 was bigger than factors humus and lignin, which means protein was less important than other factors in determining characteristic genera in SMS-conditioning groups. The relationship between protein and axis 2 mainly explained the enrichment of some peculiar genera in ABS, such as *Papillibacter*, *Saccharofermentans*, and *Parvimonas* (see Additional file 2).

## Discussion

### SCFAs production improvement in straw-conditioning WAS fermentation

Straw-conditioning was not only a better conditioning method than SMS-conditioning in this study, but also showed great advantages when comparing with related literatures [8, 19, 20]. The maximum SCFAs yield in this study was 499.6 mgCOD/gVSS, which was much higher than the SCFAs yield obtained from WAS conditioned by bagasse (360 mgCOD/gVSS) [19]. Although the SCFAs yields obtained by adding rice (520 mgCOD/gVSS) [8] and perennial ryegrass (about 528 mgCOD/gVSS) [20] were slightly higher than this study, the addition rate of carbohydrate substrates (C/N ratio 20/1) was much greater than this study (C/N ratio about 12/1). So, this study was more economically feasible when considering operation costs.

Shortening SRT will not only increase the OLR and reduce the cost but it may also affect the SCFAs production, because functional communities and their structures are closely linked to OLR. As for now, most semi-continuous experiments of WAS fermentation were conducted with a SRT of more than 8 d [26]. But in this study, when SRT was shortened to 5 d, the straw-conditioning groups still showed remarkable volumetric SCFAs productivity of 1.83 kgCOD/(${\text{m}}_{\text{reactor}}^{3} \cdot {\text{d}}$), which was nine times higher than the maximum volumetric SCFAs production of 0.2 kgCOD/(${\text{m}}_{\text{reactor}}^{3} \cdot {\text{d}}$) reported in the literatures [26].

### Synergistic effects of ARs and WAS in conditioning systems

It was observed in semi-continuous fermentation experiments that AR-conditioning (especially straw-conditioning) strongly enhanced the release and conversion of proteins in WAS. This phenomenon has proven that intensive synergistic effects existed between ARs and WAS, which well coincided with the study of Feng et al. [27]. In batch fermentation tests, it was further confirmed that the enriched bacteria in AR-conditioning co-digestion were more efficient on WAS conversion and SCFAs production even in the absence of carbohydrate substrates, compared with bacteria formed in BL. Moreover, fermentation efficiencies of straw-conditioning groups were apparently superior to those in SMS-conditioning groups with SCFAs production.

The significant difference in microbial community structures related to SCFAs production was revealed by subsequent sequencing analysis of straw-conditioning systems and SMS-conditioning systems. On one hand, the enrichment of hydrolyzing and acidifying bacteria led to an enhanced conversion from carbohydrate and protein to SCFAs in co-digestion process of WAS and AR. Hydrolysis is the most limited step for organic release and subsequent bioconversion of WAS. Straw-conditioning could promote the enrichment of bacteria secreting hydrolysis enzymes compared to BL without extra carbon addition. In straw-conditioning groups, *Lysinibacillus* was the most abundant genus which was proven to be able to secret α-chymotrypsin (an important proteolysis enzyme) and oxidize various amino acids [28]. It was reported that some species of *Lysinibacillus* were used in bioaugmentation to enhance anaerobic fermentation of food wastewater [29]. On the other hand, the characteristic genera with acid-producing abilities primarily enriched in straw-conditioning groups resulted in high SCFAs production with an enhanced conversion of carbohydrate and protein. For example, the enriched genera *Clostridium* IV (also called *Clostridium leptum*), *Xylanibacter*, *Parabacteroides*, *Clostridium sensustricto*, and *Anaerophaga* were proven to be capable of producing acids from various sugars, including all kinds of hexoses and pentoses and even some complex polysaccharides like cellulose, xylan [30–34].

In SMS-conditioning groups, the relative dominant genera like *Petrimonas*, *Levilinea*, and *Proteiniphilum* were enriched, which were capable of utilizing a wide range of sugars [35, 36] and peptone [37]. However, the relative abundance of these genera in the whole functional genera was evidently lower than straw-conditioning samples. These enriched bacteria in SMS co-digestion system were not reported to secret high efficient hydrolytic enzymes which can contribute to WAS utilization. That explained the relatively weak ability of bacteria acclimated in SMS-conditioning systems in WAS hydrolysis and acidification. The different synergistic effects induced by different types of ARs were the main reasons for their significantly different promoting effects, which were substantively determined by functional bacteria structure linking to AR conditions.

### Microbial communities and functions evolved by different ARs conditioning

It has been proven that conditioning with different types of ARs would induce distinct community structures. The link between the functional communities and their targeted products should be well understood in order to screen suitable carbohydrate substrates for WAS co-digestion. It is necessary to further understand what specific ingredients in ARs caused the enrichment of different functional bacteria. This question has been preliminarily answered by the results of CCA between characteristic microbial genera in AR-conditioning systems and ARs components. High contents of cellulose, hemicelluloses and their hydrolysate, low content of lignin and humus would be beneficial for the enrichment of bacteria with strong hydrolysis and acidification abilities (i.e., the dominant genera in straw-conditioning groups). This conclusion could be supported by the following evidences:

The characteristic genera in straw-conditioning groups included various cellulose-decomposing genera, such as *Parabacteroides, Xylanibacter, Clostridium* XI [38–40]. The genus *Lysinibacillus* with the capability of utilizing protein instead of carbohydrate widely existed in the anaerobic fermentation systems of lignocellulosic feedstocks, like corn stalk silage, straw-based vermicompost, and olive mill waste [41–43]. It hinted that these enriched genera were symbiotically connected with the simulation of celluloses and hemicelluloses in straws.

However, different genera were enriched in SMS-conditioning groups as a result of constituent content change. On one hand, the content of cellulosic substrates in SMSs was lower than straws. The total percentage of cellulose, hemicelluloses, and soluble carbohydrate were 82.7 and 83.8 % in CS and RS, but only 56 and 38.2 % in LES and ABS. On the other hand, abundant content of recalcitrant ingredients, like humus and lignin, is present in SMSs. The content of humus and lignin in SMSs was about 5.0-fold more than straws. The humus can barely be further degraded in anaerobic fermentation [44]. Lignin not only was extremely difficult to be degraded, but also hindered the contact between cellulose and microbial enzymes [22]. The presence of humus and lignin further reduced the cellulose accessibility, resulting in the formation and enrichment of specific communities. Therefore, the available cellulose content and recalcitrance impurities content in carbon conditioning to WAS played a crucial role in functional genera enrichment. ARs with high cellulose accessibility were more suitable as external carbohydrate substrates for WAS co-digestion.

## Conclusion

Conditioning by ARs significantly enhanced the hydrolysis and acid production performance of fermentation systems, which achieved considerable SCFAs yields and producing rates even under high organic loads. Significant differences in hydrolysis and acid-producing performance between two types of ARs were observed. Straws had a greater promoting effect, and were much more suitable as carbohydrate substrates compared with SMSs. High-throughput sequencing analysis revealed significant microbial community shifts induced by different AR-conditioning methods. Genera such as *Lysinibacillus, Clostridium IV*, and *Xylanibacter* were dominant in straw-conditioning groups, while totally different genera as *Proteiniphilum, Petrimonas*, and *Levilinea* were prevalent in SMS-conditioning groups. The differences in functional microbial bacteria enrichment were determined by the cellulose accessibility of ARs. The microbial communities in straw-conditioning systems were substantiated to have better SCFAs-producing abilities than those in SMS-conditioning systems, which explained the better promoting effects of straws.

## Methods

### Properties of WAS and ARs

The source and main characteristics of WAS used in this study were listed in an additional word file (see Additional file 4). The VSS concentration of WAS was controlled at 14.0 g/L to reduce the differences between batches. The C/N ratio of raw WAS was 5.9.

Two types (two kinds for each type) of ARs were used in this study, namely corn straw (CS), rice straw (RS), lentinus edodes substrate (LES), and agaricus bisporus substrate (ABS). The straws (CS and RS) were obtained from farmland in the suburb of Harbin, and the SMSs (LES and ABS) were obtained from Shuangcheng Mushroom Cultivation Base (Harbin, China). In order to improve the anaerobic digestibility, four ARs were pretreated as previously reported [17]. The specific pretreatment procedure was as follows: Initially, the ARs were dried in the oven at 70 °C until they gained constant weight. They were then chopped and milled to 2–10 mm, followed by immersion in 2 % NaOH solution at 85 °C for 1 h (by the ratio of 1 g:10 ml). Then they were dried and milled again, and stored at room temperature prior to test. The chemical composition of pretreated ARs is shown in Table 3. Volatile solid (VS) weight, instead of actual weight, was used as the basic measurement and calculation unit of ARs to exclude the influence of inactive ingredients.

**Table 3:** Characteristics of pretreated ARs

Parameter	CS	RS	LES	ABS
Volatile solid (VS) (mg/gTW)^a^	820.1	700.3	740.7	330.2
Total organic carbon (mgC/gVS)^b^	462.3	485.4	512.1	484.2
C/N ratio^c^	81.0	292.7	51.1	14.4
Cellulose (%)	50.2	56.1	44.7	16.8
Hemicellulose (%)^b^	24.6	13.4	15.5	21.2
Lignin (%)^b^	4.1	2.2	13.9	8.4
Soluble carbohydrate (%)^b^	4.8	6.6	4.5	7.8
Total protein (%)^b^	2.9	0.8	4.6	6.9
Humus (%)^b^	0.2	0.3	6.4	12.6
Other organic component (%)^b^	13.2	17.7	10.4	16.3

^a^mg volatile solid per g total weight; ^b^percentage in the volatile solid; ^c^ratio of total organic carbon and total nitrogen

### Semi-continuous fermentation experiment

**Fig. 8:**
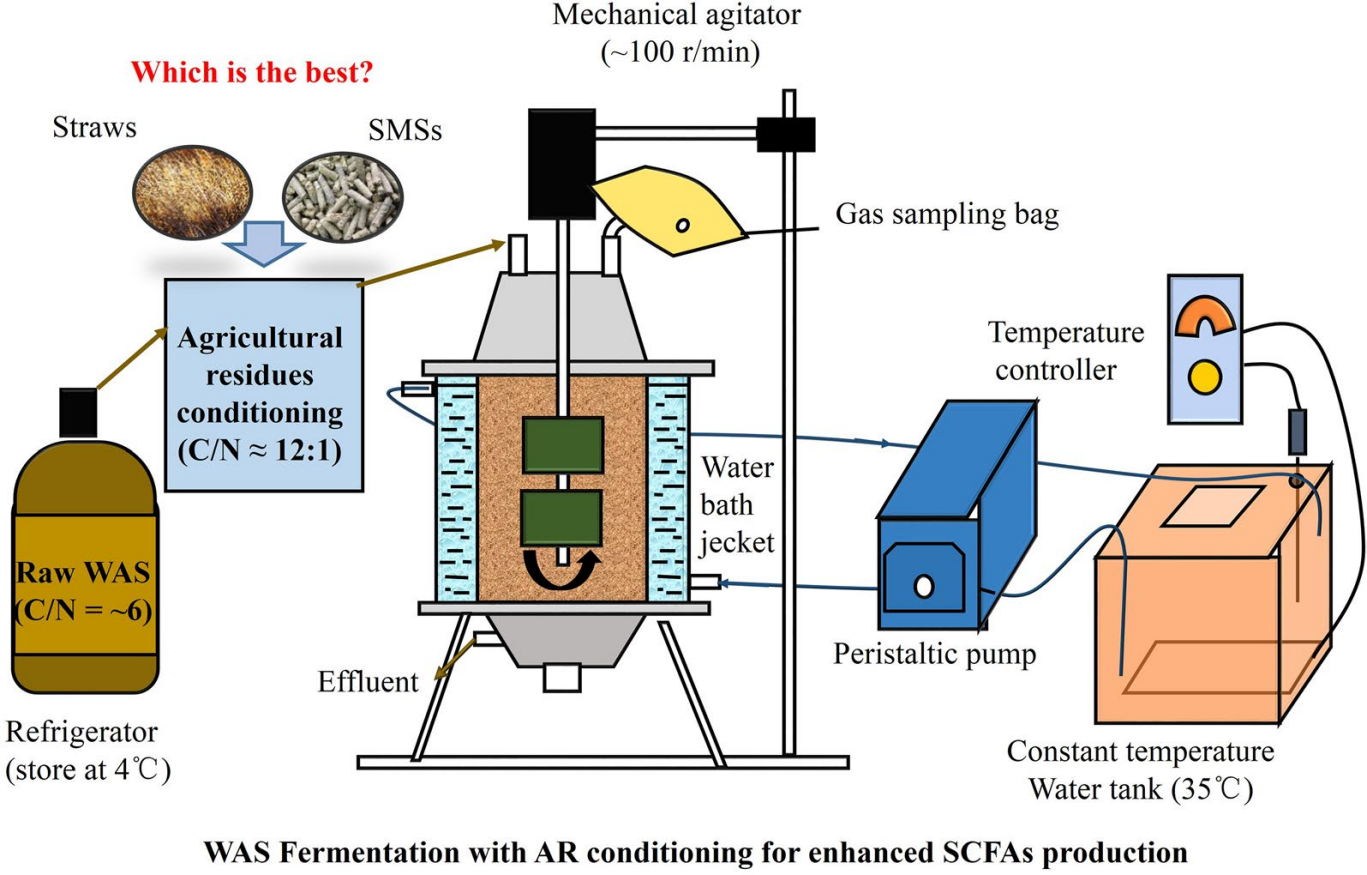
The schematic diagram of semi-continuous fermentation system. This is the *schematic diagram* of semi-continuous fermentation system used in this study. The fermentation reactor consisted of a pair of concentric plexiglass cylinders with 120 and 160 mm diameters, respectively. The inner cylinder, with a working volume of 2 L, was used for WAS fermentation, and the outer cylinder served as water jacket for temperature maintenance

Five continuously stirred-tank reactors (CSTR) (Fig. 8) [four conditioning tests with CS, RS, LES, ABS, and one blank test (indicated as BL)], with a working volume of 2 L, were used to investigate the SCFAs production performance of WAS conditioned with different types of ARs.

All reactors were operated under mesophilic alkaline anaerobic conditions, at an influent pH adjusted to 10.0 ± 0.5. In conditioning groups, the ARs addition ratio was set at 1:2 (VS_AR_:VSS_WAS_). Reactors were initially filled with feed stocks and started up with batch mode (without feeding) to enhance the preliminary acclimatization of fermentation bacteria. After 10 days the reactors were operated in a semi-continuous mode. The operation procedure was divided into three stages: SRT 10 d (day 1 ~ 20), SRT 8 d (day 21 ~ 34), and SRT 5 d (day 35 ~ 55). Sampling was performed every 12 h, and the fermented sludge in the reactors was replaced with equal amount of fresh feed stock.

### Batch fermentation test

Batch fermentation tests were conducted with a series of 500-mL serum bottles, which were inoculated with sludge taken from five semi-continuous reactors after 55 days of operation, and illustrated as CSS, RSS, LESS, ABSS, and BLS, respectively. The total organic matter content of each group was controlled at 6 gVSS, in which 0.6 gVSS was inoculated sludge (inoculation ratio 10 %) and 5.4 gVSS was WAS (without conditioning). The WAS served as substrate, and it was sterilized before use in order to eliminate the interference of active microorganisms. The specific operation process was as follows: The serum bottles were filled with sterilized WAS, and then acclimated sludge was retrieved from each semi-continuous reactor and injected into the bottles immediately. The total liquid volume of the bottles was adjusted to 400 mL by adding oxygen-free distilled water, and then the bottles were sparged with nitrogen gas to remove oxygen. All bottles were capped and sealed to maintain an anaerobic atmosphere, and were finally placed in a water bath shaker with a temperature of 35 °C to promote fermentation. All the experiments were performed in triplicates, and sampling was carried out once in 12 h, and the initial SCFAs production rate was used as an indicator of acid-producing ability of the various acclimated bacteria.

### Analytical methods

TSS and VSS of sludge samples taken from both BL and AR-conditioning groups were analyzed as previously reported [3]. Prior to analyzing soluble parameters, sludge samples were centrifuged at 10,000 rpm for 10 min, then filtered through a 0.45-μm cellulose nitrate membrane filter and stored at 4 °C. The determinations of SCOD, TCOD, soluble carbohydrates, soluble proteins, ammonia (NH_4_^+^-N), and phosphorus (PO_4_^3−^-P) were performed as previously described [6]. The measurement of SCFAs was carried out by a gas chromatography (Agilent 7890, USA) as mentioned in a previous study [45]. SCFAs production was calculated as the sum of the equivalent COD value of measured acetic (HAc), propionic (HPr), n-butyric (n-HBu), iso-butyric (iso-HBu), n-valeric (n-HVa), and iso-valeric (n-HVa) acids. COD conversion factors are 1.5 gCOD/g protein, 1.06 gCOD/g carbohydrate, 1.07 gCOD/g HAc, 1.51 gCOD/g HPr, 1.82 gCOD/g HBu, and 2.04 gCOD/g HVa.

The content of cellulose, hemicellulose, and lignin in ARs was determined as previously reported [46]. Kjeldahl method was used for the determinations of total protein, total carbon (TC) and total nitrogen (TN) in WAS and ARs, and was analyzed by an elemental analyzer (Elemental Analyzer NA 2500), and the C/N ratio was calculated as TC/TN. The soluble carbohydrate and protein of ARs were extracted by water in a 50 °C water bath for 12 h, and measured by the same method with sludge filtrate. The humus substances were extracted and determined by the similar method mentioned in the literature [47].

### Statistical analysis

Statistical significance was determined by analysis of variance (ANOVA, *α* = 0.05) using IBM SPSS Statistics (version 19). PCA and CCA were carried out using Canoco software package (version 4.5).

### DNA extraction, PCR amplification, and Phylogenetic analysis

Total genomic DNA of five sludge samples (CS, RS, LES, ABS, and BL) taken from the semi-continuous reactors after 55 days operation was extracted with OMEGA Soil DNA Isolation Kit (OMEGA Bio-Tek Inc, Norcross, GA, USA) according to the manufacturer’s instructions. The quantity and quality of the extracted DNA were checked with Qubit 2.0 photometer (Life Technologies, Inc, USA). Amplicon liberates were constructed for Illumina Miseq sequencing using bacterial fused primers 341F (CCTACACGACGCTCTTCCGATCTN (barcode) CCTACGG–GNG GCWGCAG) and 805R (GACTGGAGTTCCTTGGCACCCGAGAATT-CCA (barcode) GACTACHVGGGTATCTAATCC) for the V3–V4 region of the 16S rRNA gene [48]. The barcodes were used to sort multiple samples in a single Miseq sequencing run. PCR reactions were performed in a total volume of 50 μL containing 1XPCR buffer, 0.1 mm dNTPs, 0.5 μm each primer, 0.05 U Plantinum Taq, and 10 ng of template DNA. The PCR amplification program contained an initial denaturation at 94 °C for 3 min, followed by 5 cycles of denaturing at 94 °C for 30 s, annealing at 45 °C for 20 s, and extension at 65 °C for 30 s, then followed by 20 cycles of denaturing at 94 °C for 20 s, annealing at 55 °C 20 s, and extension at 72 °C for 30 s, finally followed by a final extension at 72 °C for 5 min. Before sequencing, the PCR products of different samples were normalized in equimolar amounts in the final mixture, which was used to construct the PCR amplicon libraries. Sequencing was carried out on an Illumina Miseq, and the raw sequences data have been deposited in the NCBI Sequence Read Archive (SRA) database with the accession numbers of SRP059974.

## Additional files

10.1186/s13068-015-0369-x The mean VFAs concentrations and T-test analysis results of each operation stages in semi-continuous co-fermentation experiment.
10.1186/s13068-015-0369-x Phylogenetic classification of the 16S DNA gene sequence (relative abundance >1 % at genus levels) in the CS, RS, LES, ABS and BL.
10.1186/s13068-015-0369-x The eigenvalues of first two canonical axes and their relationships with each environmental factor.
10.1186/s13068-015-0369-x Characteristics of waste activated sludge used in the experiment.

## Abbreviations

WAS: waste activated sludge
ARs: agricultural residues
SMSs: spent mushroom substrates
SCFAs: short-chain fatty acids
SRT: sludge retention time
OLR: organic load rate
C/N ratio: carbon-to-nitrogen ratio
CS: corn straw
RS: rice straw
LES: lentinus edodes substrate
ABS: agaricus bisporus substrate
COD: chemical oxygen demand
SCOD: soluble chemical oxygen demand
CSTR: continuously stirred-tank reactor
VS: volatile solids
VSS: volatile suspended solids
TSS: total suspended solids
CCA: canonical correlation analysis
