# Erratum to: Enhanced short chain fatty acids production from waste activated sludge conditioning with typical agricultural residues: carbon source composition regulates community functions

**DOI:** 10.1186/s13068-016-0482-5

**Published:** 2016-03-16

**Authors:** Zechong Guo, Aijuan Zhou, Chunxue Yang, Bin Liang, Thangavel Sangeetha, Zhangwei He, Ling Wang, Weiwei Cai, Aijie Wang, Wenzong Liu

**Affiliations:** State Key Laboratory of Urban Water Resource and Environment, Harbin Institute of Technology (SKLUWRE, HIT), Harbin, China; College of Environmental Science and Engineering, Taiyuan University of Technology, Taiyuan, China; Key Laboratory of Environmental Biotechnology, Research Center for Eco-Environmental Sciences, Chinese Academy of Sciences, Beijing, China

## Erratum to: Biotechnol Biofuels (2015) 8:192 DOI 10.1186/s13068-015-0369-x

Following publication of the original article in *Biotechnology for Biofuels* [1], it was brought to our attention that Zechong Guo was incorrectly listed as the corresponding author. The correct corresponding authors are Aijie Wang (waj0578@hit.edu.cn) and Wenzong Liu (wzliu@rcees.ac.cn). We apologize for the inconvenience this may have caused.
