# Supplementary material for: Enhanced short chain fatty acids production from waste activated sludge conditioning with typical agricultural residues: carbon source composition regulates community functions
Source: Biotechnol Biofuels. 2015 Nov 25;8:192. doi: 10.1186/s13068-015-0369-x (PMC4660719; doi:10.1186/s13068-015-0369-x)
Supplement: Supplementary file 1 — 10.1186/s13068-015-0369-x The mean VFAs concentrations and T-test analysis results of each operation stages in semi-continuous co-fermentation experiment. [file 13068_2015_369_MOESM1_ESM.docx]

**Additional file 1**

**The mean VFAs concentrations and T-test analysis results of each operation stages in semi-continuous co-fermentation experiment.**

Data processing was applied to the results of semi-continuous experiment to eliminate the fluctuations in data before averaging and comparing the differences. The data of first several days of each operate stages (5 days for stage 1, 4 days for stage 2, 5 days for stage 3) was considered in unstable state, so they were removed. The remaining data were supposed to follow normal distribution, first standardize them to standard normal distribution (μ=0, σ=1), then remove the data with a value below -2 or above 2, repeat standardization and deletion until all data were within twice the variance, the original value corresponding to the remaining data were identified as valid data. Standardization and statistical analysis were all carried out in software IBM SPSS Statistics (version 19).

**Table 1** The mean values and T-test results of VFAs concentration in each stages

| group | SRT=10 | | | | SRT=8 | | | | SRT=5 | | | |
| --- | --- | --- | --- | --- | --- | --- | --- | --- | --- | --- | --- | --- |
|  | Mean^a^ | N^b^ | SE^c^ | Sig. | Mean | N | SE | Sig. | Mean | N | SE | Sig. |
| CS | 8743.1 | 13 | 164.8 | 0.15^d^ | 9942.7 | 10 | 1036.8 | 0.25^d^ | 9039.0 | 14 | 299.4 | 0.23^d^ |
| RS | 9044.2 | 15 | 220.1 |  | 10491.8 | 10 | 1049.2 |  | 9247.2 | 15 | 218.8 |  |
| LES | 5576.4 | 12 | 80.9 | 0.004^e^ | 6271.9 | 10 | 1223.8 | 0.51^e^ | 4859.0 | 15 | 326.5 | 0.12^e^ |
| ABS | 5880.4 | 13 | 54.6 |  | 6601.6 | 10 | 974.7 |  | 4899.0 | 15 | 423.7 |  |
| BL | 2918.6 | 15 | 72.7 | <0.01^f^ | 3508.6 | 8 | 105.8 | <0.01^f^ | 2880.1 | 12 | 106.2 | <0.01^f^ |

a the mean value of VFAs concentration; b effective data number; c standard error; d the significance of t-test between the mean value of CS and RS; e t-test significance between LES and ABS; f t-test significance between blank test and each other group
