# Supplementary material for: Enhanced short chain fatty acids production from waste activated sludge conditioning with typical agricultural residues: carbon source composition regulates community functions
Source: Biotechnol Biofuels. 2015 Nov 25;8:192. doi: 10.1186/s13068-015-0369-x (PMC4660719; doi:10.1186/s13068-015-0369-x)
Supplement: Supplementary file 2 — 10.1186/s13068-015-0369-x Phylogenetic classification of the 16S DNA gene sequence (relative abundance >1 % at genus levels) in the CS, RS, LES, ABS and BL. [file 13068_2015_369_MOESM2_ESM.docx]

**Additional file 2**

**Phylogenetic classification of the 16S DNA gene sequence (relative abundance >1% at genus levels) in the CS, RS, LES, ABS and BL**

**Table 1** Phylogenetic classification of the 16S DNA gene sequence (relative abundance >1% at genus levels) in the CS, RS, LES, ABS and BL.

| Genus | Class | Phylum | CS^a^ | RS^a^ | LES^a^ | ABS^a^ | BL^a^ |
| --- | --- | --- | --- | --- | --- | --- | --- |
| *Clostridium IV* | *Clostridia* | *Firmicutes* | **10.36** | **5.56** | 0.65 | 0.53 | 0.19 |
| *Lysinibacillus* | *Bacilli* | *Firmicutes* | **9.42** | **11.23** | 0.03 | 0.18 | 0.01 |
| *Xylanibacter* | *Bacteroidia* | *Bacteroidetes* | **4.23** | **6.18** | 0.02 | 0.03 | 0.01 |
| *Parabacteroides* | *Bacteroidia* | *Bacteroidetes* | **3.39** | **3.51** | 0.93 | 0.12 | 0.06 |
| *Clostridium sensu stricto* | *Clostridia* | *Firmicutes* | **3.13** | **1.45** | 0.77 | 0.34 | 0.18 |
| *Phascolarctobacterium* | *Negativicutes* | *Firmicutes* | **2.06** | **0.55** | 0.21 | 0.02 | 0.01 |
| *Anaerophaga* | *Bacteroidia* | *Bacteroidetes* | **2.00** | **1.93** | 0.32 | 0.24 | 0.01 |
| *Clostridium XI* | *Clostridia* | *Firmicutes* | **1.38** | **0.93** | 0.15 | 0.05 | 0.06 |
| *Cloacibacillus* | *Synergistia* | *Synergistetes* | 1.29 | 0.48 | 0.24 | 0.58 | 0.33 |
| *Bacteroides* | *Bacteroidia* | *Bacteroidetes* | 0.83 | 2.57 | 1.79 | 0.08 | 0.01 |
| *Kurthia* | *Bacilli* | *Firmicutes* | 0.06 | 1.33 | 0 | 0 | 0 |
| *Tetrasphaera* | *Actinobacteria* | *Actinobacteria* | 0.88 | 1.27 | 0.75 | 1.11 | 0.05 |
| *Ruminococcus* | *Clostridia* | *Firmicutes* | 0.30 | 1.12 | 0.57 | 0.09 | 0.01 |
| *Prevotella* | *Bacteroidia* | *Bacteroidetes* | 1.17 | 0.70 | 0.01 | 0 | 0 |
| *Proteiniphilum* | *Bacteroidia* | *Bacteroidetes* | 0.06 | 0.05 | **16.80** | **11.41** | 3.64 |
| *Petrimonas* | *Bacteroidia* | *Bacteroidetes* | 0.06 | 0.21 | **7.96** | **2.90** | 2.77 |
| *Levilinea* | *Anaerolineae* | *Chloroflexi* | 0.06 | 0.06 | **1.90** | **2.58** | 0.02 |
| *Anaerorhabdus* | *Bacteroidia* | *Bacteroidetes* | 0.03 | 0.68 | 1.74 | 0.02 | 0.08 |
| *Longilinea* | *Anaerolineae* | *Chloroflex* | 0.14 | 0.16 | 1.36 | 0.89 | 0.09 |
| *Proteiniclasticum* | *Clostridia* | *Firmicutes* | 0.06 | 0.10 | 1.36 | 0.42 | 0.25 |
| *Sedimentibacter* | *Clostridia* | *Firmicutes* | 0.06 | 0.07 | 1.33 | 0.65 | 0.33 |
| *Flavonifractor* | *Clostridia* | *Firmicutes* | 0.07 | 0.07 | 1.31 | 0.06 | 0.09 |
| *Proteocatella* | *Clostridia* | *Firmicutes* | 0.03 | 0.10 | 1.28 | 0.03 | 0.02 |
| *Oscillibacter* | *Clostridia* | *Firmicutes* | 0.95 | 0.89 | 1.22 | 0.08 | 0.03 |
| *Thermoflavimicrobium* | *Bacilli* | *Firmicutes* | 0.04 | 0.01 | 0.11 | 14.65 | 1.20 |
| *Parvimonas* | *Clostridia* | *Firmicutes* | 0 | 0 | 0.01 | 2.56 | 0.27 |
| *Papillibacter* | *Clostridia* | *Firmicutes* | 0.06 | 0.1 | 0.13 | 1.7 | 0.07 |
| *Acinetobacter* | *Gammaproteobacteria* | *Proteobacteria* | 0.09 | 0.05 | 0.45 | 1.69 | 0.52 |
| *Guggenheimella* | *Clostridia* | *Firmicutes* | 0.03 | 0.03 | 0.01 | 2.35 | **15.08** |
| *Pseudomonas* | *Gammaproteobacteria* | *Proteobacteria* | 0.01 | 0 | 0.04 | 0.08 | **6.27** |
| *Saccharofermentans* | *Clostridia* | *Firmicutes* | 0.13 | 0.14 | 0.37 | 7.30 | **5.63** |
| *Tissierella* | *Clostridia* | *Firmicutes* | 0.03 | 0.04 | 1.91 | 2.00 | **4.75** |
| *Fluviicola* | *Flavobacteria* | *Bacteroidetes* | 0.03 | 0.03 | 0.03 | 0.01 | **4.44** |
| *Erysipelothrix* | *Erysipelotrichia* | *Firmicutes* | 0.06 | 0.01 | 0.01 | 0.02 | **3.82** |
| *Pusillimonas* | *Betaproteobacteria* | *Proteobacteria* | 0 | 0.01 | 0 | 0 | **1.73** |
| *Meniscus* | *Sphingobacteria* | *Bacteroidetes* | 0.05 | 0.12 | 0.52 | 0.14 | **1.63** |
| *Truepera* | *Deinococci* | *Deinococcus-Thermus* | 0 | 0 | 0 | 0 | **1.56** |
| *Paracoccus* | *Alphaproteobacteria* | *Proteobacteria* | 0.14 | 0.16 | 0.17 | 0.34 | **1.34** |
| *Wandonia* | *Flavobacteria* | *Bacteroidetes* | 0.01 | 0.01 | 0 | 0 | **1.32** |
| *Ornithobacterium* | *Flavobacteria* | *Bacteroidetes* | 0 | 0.01 | 0.10 | 0.11 | **1.20** |
| *Desulfomicrobium* | *Deltaproteobacteria* | *Proteobacteria* | 0 | 0 | 0 | 0 | **1.17** |
| *Gracilimonas* | *Sphingobacteria* | *Bacteroidetes* | 0 | 0 | 0 | 0 | **1.09** |
| *TM7_genera_incertae_sedis* | *TM7* |  | 0.45 | 0.53 | 0.42 | 0.49 | **1.08** |
| ^a^ relative abundance of genus in each group, %  The characteristic genera of straw-conditioning groups, SMS-conditioning groups and BL was bolded. | | | | | | | |
