# Supplementary material for: Enhanced short chain fatty acids production from waste activated sludge conditioning with typical agricultural residues: carbon source composition regulates community functions
Source: Biotechnol Biofuels. 2015 Nov 25;8:192. doi: 10.1186/s13068-015-0369-x (PMC4660719; doi:10.1186/s13068-015-0369-x)
Supplement: Supplementary file 3 — 10.1186/s13068-015-0369-x The eigenvalues of first two canonical axes and their relationships with each environmental factor. [file 13068_2015_369_MOESM3_ESM.docx]

**Additional file 3**

**The eigenvalues of first two canonical axes and their relationships with each environmental factor**

**Table 1** The eigenvalues of first two canonical axes and their relationships with each environmental factor

|  | Axis 1 | Axis 2 |
| --- | --- | --- |
| Elgenvalues | 0.784 | 0.320 |
| Cumulative percentage variance | 66.9% | 94.2% |
| Lignin | 0.9573 | -0.2175 |
| Humus | 0.9566 | -0.2909 |
| Hemicellulose | -0.8155 | 0.3132 |
| Cellulose | -0.9873 | 0.1448 |
| Soluble carbohydrate | -0.9147 | 0.1306 |
| Protein | 0.8159 | -0.5716 |
