# Supplementary material for: Enhanced short chain fatty acids production from waste activated sludge conditioning with typical agricultural residues: carbon source composition regulates community functions
Source: Biotechnol Biofuels. 2015 Nov 25;8:192. doi: 10.1186/s13068-015-0369-x (PMC4660719; doi:10.1186/s13068-015-0369-x)
Supplement: Supplementary file 4 — 10.1186/s13068-015-0369-x Characteristics of waste activated sludge used in the experiment. [file 13068_2015_369_MOESM4_ESM.docx]

**Additional file 4**

**Characteristics of waste activated sludge used in the experiment**

The waste activated sludge (WAS) used in this study was collected from the secondary sedimentation tank at the Taiping Municipal Wastewater Treatment Plant (Harbin, China), and was concentrated by settling for 24 h, screened with a 1 mm sieve to remove impurities and then stored at 4 °C. The VSS of WAS were adjusted to 14.0 g/L with tap water prior to be used. The main characteristics of WAS were shown in Table S1.

**Table 1** Characteristics of waste activated sludge used in the experiment

| Parameter | Raw WAS value^a^ |
| --- | --- |
| pH | 6.57 ± 0.15 |
| Total suspended solids (TSS) (g/L) | 18.92 ± 0.81 |
| Volatile suspended solids (VSS) (g/L) | 14.00 |
| Soluble chemical oxygen demand (SCOD) (mg/L) | 356 ± 52 |
| Total chemical oxygen demand (TCOD) (mg/L) | 20790 ± 216 |
| Soluble carbohydrate (mg/L) | 67 ± 17 |
| Soluble protein (mg/L) | 21 ± 8 |
| Volatile fatty acids (VFAs) (as mgCOD/L) | 215± 39 |
| Carbon to nitrogen mass (C/N ratio) | 5.9± 0.2 |

^a^ average ± standard deviation.
